# Development of a User-Adaptable Human Fall Detection Based on Fall Risk Levels Using Depth Sensor

**DOI:** 10.3390/s18072260

**Published:** 2018-07-13

**Authors:** Yoosuf Nizam, Mohd Norzali Haji Mohd, M. Mahadi Abdul Jamil

**Affiliations:** 1 Biomedical Engineering Modeling and Simulation (BIOMEMS) Research Group, Faculty of Electrical and Electronic Engineering, Universiti Tun Hussein Onn Malaysia, 86400 Parit Raja, Batu Pahat, Johor, Malaysia; he140090@siswa.uthm.edu.my (Y.N.); mahadi@uthm.edu.my (M.M.A.J.); 2 Embedded Computing Systems (EmbCos) Research Group, Faculty of Electrical and Electronic Engineering, Universiti Tun Hussein Onn Malaysia, 86400 Parit Raja, Batu Pahat, Johor, Malaysia; 3 Computer Signal, Imaging and Intelligent (CSII) Research Group, Faculty of Electrical and Electronic Engineering, Universiti Tun Hussein Onn Malaysia, 86400 Parit Raja, Batu Pahat, Johor, Malaysia

**Keywords:** falls, human fall, assistive living, daily activities, fall risk level

## Abstract

Unintentional falls are a major public health concern for many communities, especially with aging populations. There are various approaches used to classify human activities for fall detection. Related studies have employed wearable, non-invasive sensors, video cameras and depth sensor-based approaches to develop such monitoring systems. The proposed approach in this study uses a depth sensor and employs a unique procedure which identifies the fall risk levels to adapt the algorithm for different people with their physical strength to withstand falls. The inclusion of the fall risk level identification, further enhanced and improved the accuracy of the fall detection. The experimental results showed promising performance in adapting the algorithm for people with different fall risk levels for fall detection.

## 1. Introduction

Daily living assistance is very often needed for many people in today’s aging population, including disabled, overweight, obese and elderly people. The main purpose of assistive technology is to provide better living and health care to those in need, especially elderly people who live alone. It is mainly aimed at allowing them to live in their own home independently as long as possible without having to change their life style. 

To provide better living for the elderly and those with special needs, it is important to have continuous human monitoring systems in their home to inform the health care representatives of any emergency attendance. Among such monitoring systems, fall detection systems are of increasing interest since statistics [1,2] have shown that falling is the main reason of injury-related death for seniors aged 79 [3,4] or above, and it is the second most common cause of injury-related (unintentional) death for all ages [5,6]. Furthermore, falling is the biggest threat among all other incidents to the elderly and those people who are in need of support [3,7,8,9,10,11,12,13,14,15,16]. Accordingly, for elderly people, falls can have severe consequences, especially if not attended to in a short period of time [17]. Human falls also represent the main source of morbidity and mortality among elderly [18].

Related studies have employed different approaches including wearable sensors, non-wearable sensors and vision-based sensors to realize fall detection [19,20,21,22,23,24]. Wearable devices such as belts and other non-wearable devices, such as floor vibration sensors, are very cheap and easy to setup [25,26,27]. On the other hand, such sensors are prone to generating high false alarms and therefore are not reliable. As far as vision-based devices are concerned, they are very accurate in classifying falls from other activities of daily life. Even though vision-based devices are expensive and more difficult to setup than wearable-based devices, they are reliable and generate less false alarms. There are also studies that used depth sensors for fall detection to overcome some of the drawbacks of camera-based devices [28]. It is also to be noted that the nature of falls differ from person to person depending on their physical conditions, including gait weakness, diseases and balancing problems. These differences in the nature of falls and people with higher chances of falls can be related to their fall risk levels. To our knowledge, there is no study that consider the fall risk levels for fall detection. Identification of the fall risk level of the user, can help to improve and adjust fall detection to adapt the procedure to people of different fall risk levels, such as intensive monitoring for those with a higher risk of falls and moderate scanning for people with less chance of falls. This paper proposes a reliable human fall detection using a depth sensor (Kinect sensor) which is adaptive to the user’s physical condition (fall risk level or how susceptible to unintentional fall). The rest of this paper is organized as follows. The next section highlights some of the related works, followed by the proposed method for fall detection using Kinect sensors. Finally, the experimental results with a brief discussion are presented before concluding the paper.

## 2. Related Works

Since this work focuses on the use of depth information to classify human activities for fall detection, this section will review selected studies that have based their fall detection on depth sensors. One study proposed as an unobtrusive fall detection system, was running in real time with a novel algorithm, proposed by Praveen Kumar et al., using a Kinect sensor. The proposed novel fall detection algorithm is made up of several steps. A fall from sitting or standing is confirmed if the body motion gets involved in the Y or Z coordinate. A sudden fall is also monitored by the time scale of the fall from the human towards the respective axis [29].

Another fall detection algorithm method proposed by Yang et al., uses shape analysis of depth images from Kinect for indoor environment. A median filter is used to extract the background and target from the depth images. The silhouette of the moving object in the scene is extracted by the background frames subtraction method and the floor plane by using a v-disparity map. The shape characteristics of moving individuals are described by an ellipse. The distance of the centroids and the angles between the ellipses are computed, and fall is detected when the distance and angle between the ellipses and floor plane are lower than some threshold. Experimental results show that the proposed method is accurate in detecting fall events [30].

The head detection algorithm from the depth video using the Kinect sensor and its application for human fall detection is proposed by Nghiem et al. The reason for head detection is to distinguish the human subject from other objects in the scene and it gives useful information to better identify human falls. A human is recognized using a modified Histogram of Oriented Gradient (HOG). The proposed algorithm first detects possible head position and then based on the head position, the subject is recognized by detecting head and shoulders. The fall detection is based on the vertical speed and the distance from the ground to head and the centroid. The proposed algorithm first detects the head position and calculates its vertical speed and checks if the falling condition is satisfied, which is a speed threshold of 2 m/s and a height threshold of 0.5 m [31].

The use of depth data from a Kinect sensor was introduced to propose a new technique to detect fall by Planinc and Kampel. As opposed to the current emergency systems for elderly, which contain a wearable sensor or a button-based device to call for assistance, this paper first presents three different non-invasive-based approaches. Fall is detected, if the major orientation of the person is parallel to the floor and the height of the spine is near the floor. The developed algorithm using the Kinect depth sensor was evaluated against state-of-the-art approaches using 2D sensors or microphones. The results after improving the tracking of the skeleton when the person leaves the frame, show an accuracy of 87.5% with 100% precision and 77.5% recall [32].

Bian et al. presented a method for fall detection based on the distance between human skeleton joints to the floor, and the joint velocity. The velocity of the joint hitting the floor is used to distinguish the fall accident from sitting or lying down on the floor. Fall is detected if the distance between the head and floor is lower than the recover threshold for a certain period of time. The distance threshold used for the three joints and the recover threshold used for the head is adaptive to the height of the person [33].

In another study, a mobile robot system was introduced, which follows a person and detects when the person has fallen using a Kinect sensor. The use of the mobile robot to follow the elderly people can solve the coverage limitation of using a fixed Kinect sensor. Gesture and speech recognition is implemented to provide human-robot-interaction. A fall is detected by thresholding the distance between key joints (head, shoulder, hip center and ankles) to the floor, if the floor is visible or detected. If the floor plane is not detected then a second algorithm is used which depends on the skeleton coordinate system. The second algorithm detects a fall if the y-coordinates of the joints mentioned are less than a given threshold. The experiments were conducted in a real indoor environment with different lighting conditions [34]. 

A fuzzy inference-based system using Kinect and a device with an accelerometer and a gyroscope is shown to achieve reliable fall detection. The use of an accelerometer and a video-based approach complement each other for different situations, for example, a wearable sensor might not be comfortable during changing clothes, washing etc. In such a situation the system relies on the Kinect camera only. The experimental results showed the high accuracy of detection and effectiveness of the system [35]. 

The method proposed by Ma et al., uses a combination two computer vision approach; where fall characterization is done using a shape-based approach and a machine learning classifier is used to identify human fall from other activities. At first, human silhouette is extracted from depth images. Adaptive Gaussian Mixture Model (GMM) is used for human segmentation from the background. The second step involves finding of the features of the detected subject. Kinect sensor was placed at a height of 1.5 m and features were extracted using c++ for an experimental dataset. Experimental results show that human silhouettes are extracted even with no light conditions and the proposed approach is able to gain an accuracy of 86.83% using a single camera [36].

A privacy-preserving fall detection method proposed by Gasparrini et al., used raw data directly from the sensor. The data were analyzed, and the system extracted the elements to classify all the blobs in the scene through the implemented solutions. A fall is detected if the depth blob associated to a person is near to the floor [28].

## 3. Methods

The method proposed in this study used the Microsoft Kinect Sensor for the classification of daily life activities to identify any human fall event. The basic components of fall detections are the changes in speed with direction (velocity) and height (changes or drop to floor level), which is applied in different orders depending on the fall risk levels of the subject. Fall risk level of the subject is a measure of physical weakness or any difficulties the subject is facing during their daily life activities that may have higher chances of falls than other normal elderly people. Fall risk level is measured using a few widely-used fall risk assessment parameters. The following Section 3.1 and 3.2 will describe the parameters or the variables employed to derive activity classification parameters and the procedures used to find the fall risk levels, respectively. Section 3.3 will demonstrate the proposed fall detection algorithm which will detect any unintentional human fall using the variables described in previous sub-sections.

### 3.1. Classification Parameters

The proposed algorithm uses the floor plan and the generated skeleton coordinates as a basis for the distance or height calculation. The sensor is placed at about a height of 3 feet from the floor for a front view configuration. To calculate the distance between any joint and the floor, the joint coordinates and floor plane equation can be applied to the following Equation (1).

(1) $$\mathrm{Height}\;\left(H\right)=\;\frac{\left|\mathrm{Ax}\;+\;\mathrm{By}\;+\;\mathrm{Cz}\;+\;D\right|\;}{\surd \left(A^{2}\;+{\;B}^{2}\;+{\;C}^{2}\right)\;}$$

where: x, y and z are the coordinates of the joints. A, B, and C are the x, y, and z values extracted from the floor clipping vector contained in the skeleton data. Here, D is simply the height of the camera from the floor in meters.

**Figure 1:**
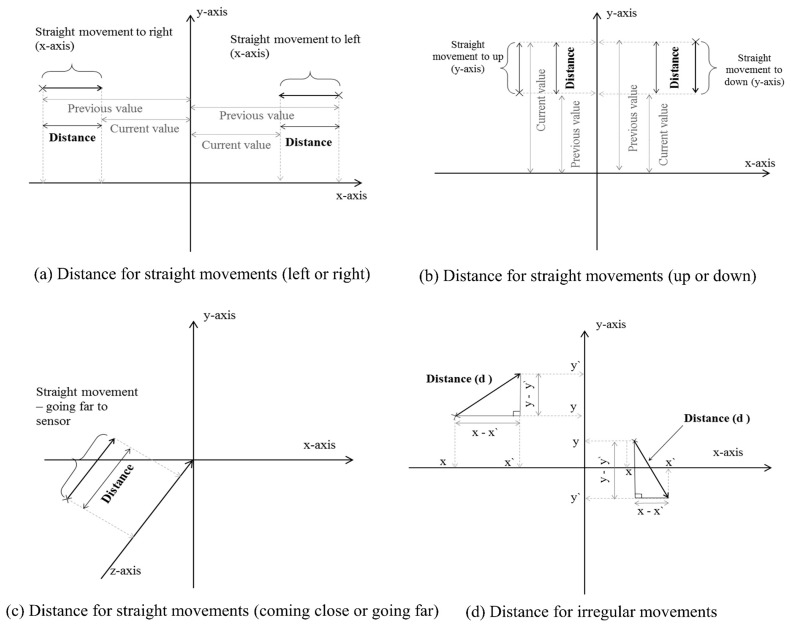

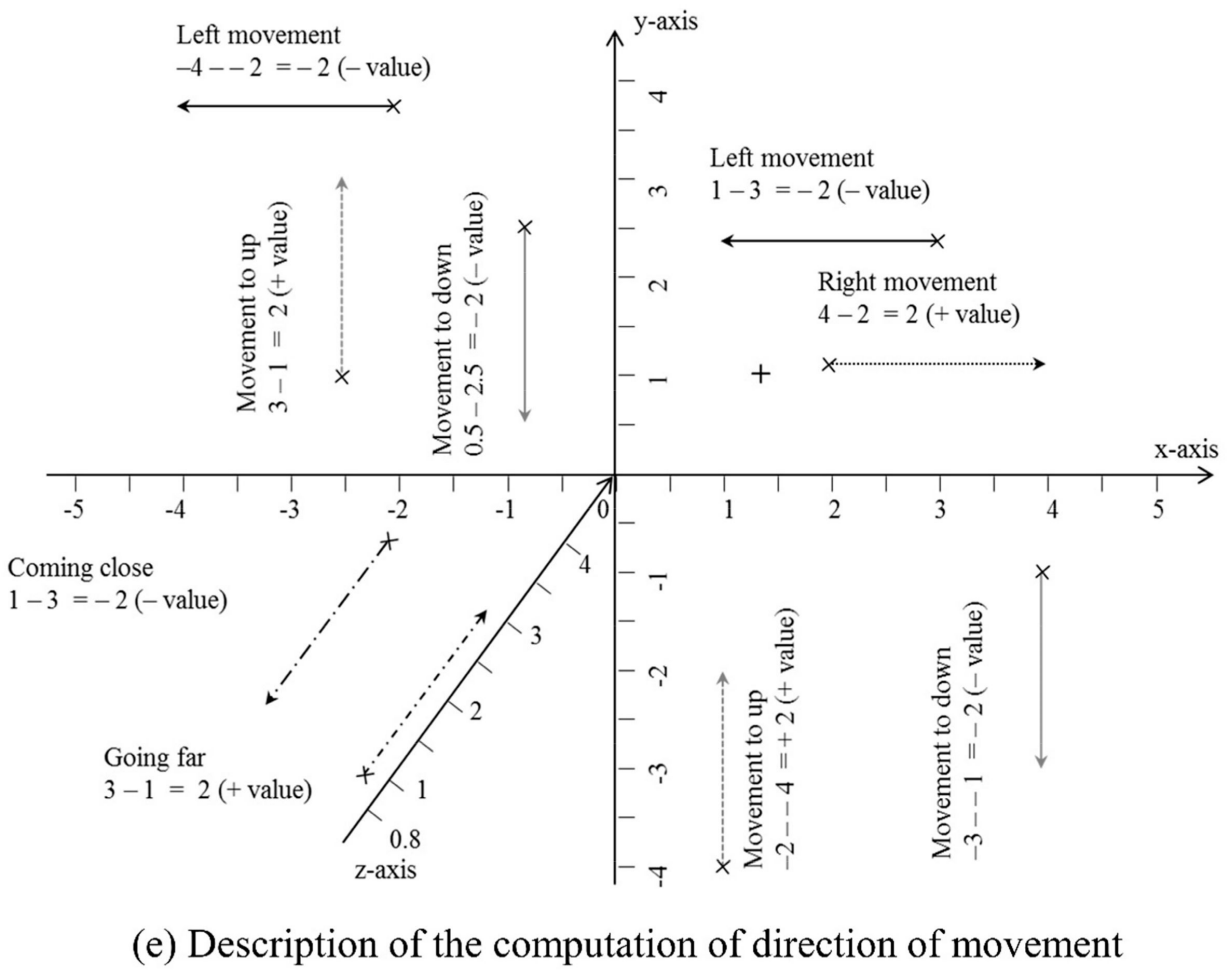
The description for the calculation of the distance for velocity and direction of movement with the coordinate system.

Velocity is calculated after determining the direction of the movement, which is described in Figure 1e. The distance travelled for the movement is divided by the time taken for the movement to calculate the speed, as shown in Equation (2). The time taken for the movement is 1/15 s, because the sensor generates 30 frames per second and the joint position is taken after skipping one frame (time for two frames). For the magnitude component of velocities, the same concept as for speed (Equation (2)) is used except that the speed for the shoulder center and hip center is also calculated together with direction.

(2) $$\mathrm{Speed},\;\left(\mathrm{Magnitude}\;\mathrm{component}\;\mathrm{of}\;\mathrm{velocity}\right)=\;\frac{D_{c}\;-{\;D}_{p}}{t_{c}\;-{\;t}_{p}}\;\mathrm{Meter}/\sec\mathrm{ond}$$

where D_c_ is the Current Distance (current joint coordinate), D_p_ is the Previous Distance (previous joint coordinate), t_c_ is the current time in seconds and t_p_ is the previous time in seconds.

If the direction is vertical (irregular) to any side (any axis), the distance travelled, cannot be simply calculated by subtracting the position between two frames on any axis, because the changes are not on the axis and so if the changes are considered on the axis, then the distance will be less than the actual distance travelled. Equation (3), is used to calculate such irregular distances. Once distance for any irregular movement is calculated, the magnitude part of the velocity can be calculated by using Equation (2). The distance computation is graphically described in Figure 1a–d.

(3) $$D,\;\left(\mathrm{distance}\;\mathrm{for}\;\mathrm{irregular}\;\mathrm{movements}\right)=\;\sqrt{\left({\left(y\;-\;y`\right)}^{2}+{\left(x\;-\;x`\right)}^{2}\right)}$$

### 3.2. Fall Risk Factors

**Figure 2:**
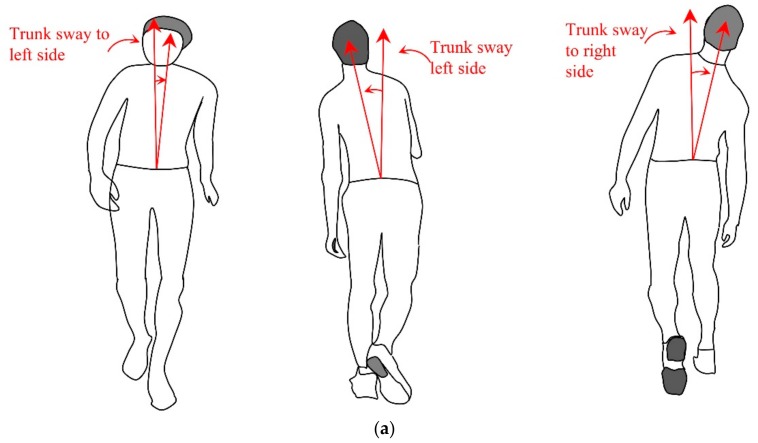

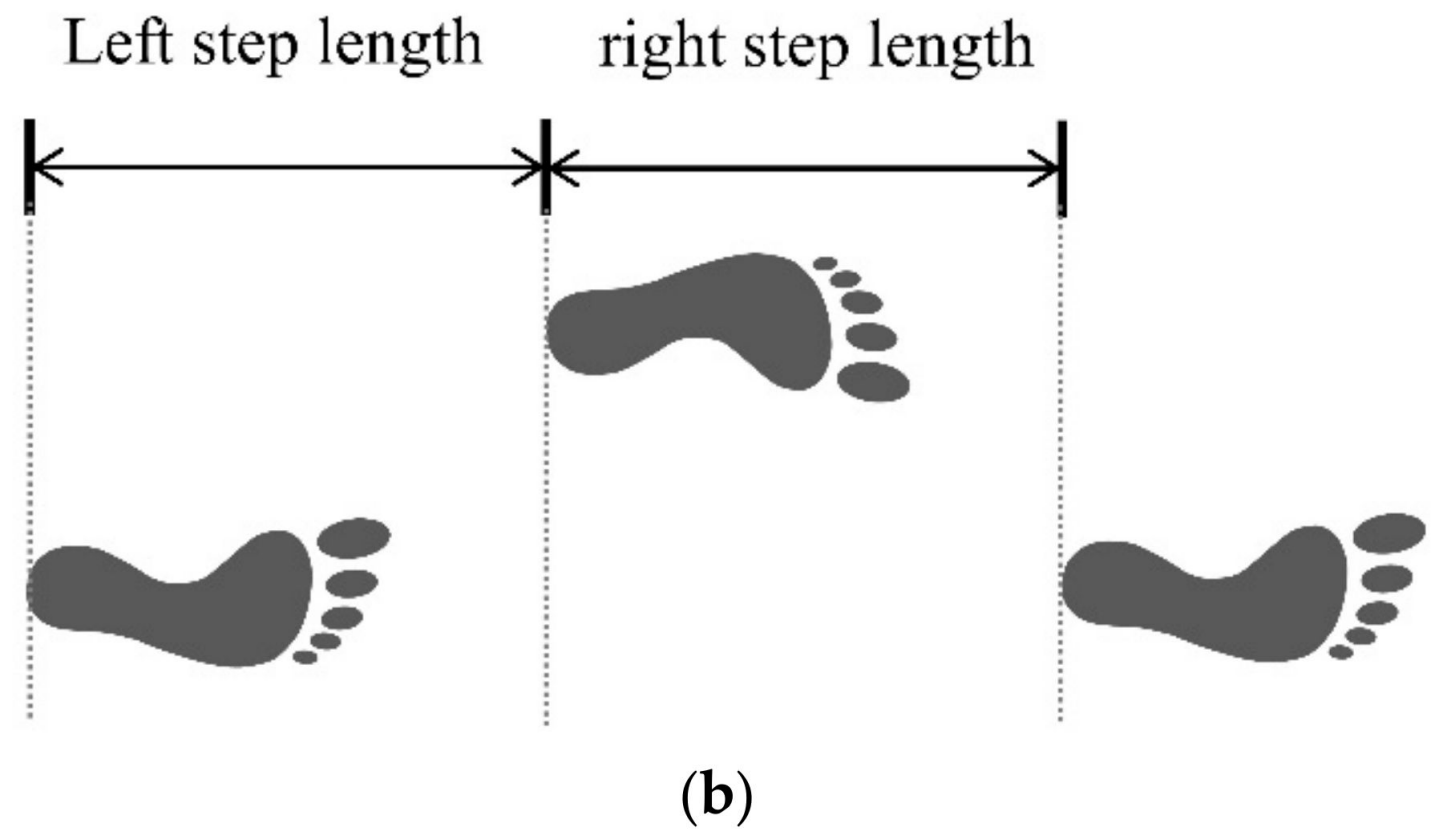
Illustration of trunk sway and step length. (**a**) Trunk sway; (**b**) Step length.

Fall risk factors are used to identify any abnormality from the movements and identify any existence of physical weakness that can easily cause another fall event. The measures considered are the step_symmetry, trunk_sway and spread_arm. The step_symmetry, is the estimate of the step inequality which can be realized by measuring the left and the right step lengths. Step length is the distance between the left and right step, which can be measured using the x-axis or z-axis coordinates depending on the direction of the movement. If the direction of the movement is on the x-axis then the following Equation (4), is used to compute the Step_symmetry, and if the direction of the movement is on z-axis then simple z-values are used instead of x-values in the equation. Left and right step length and trunk sway is illustrated in Figure 2.

(4) $$\;\;\mathrm{Step}\_\mathrm{symmetry}\;={\left(R\_{\mathrm{foot}}_{x}-L\_{\mathrm{foot}}_{x}\right)}_{\mathrm{PF}}-{\left(R\_{\mathrm{foot}}_{x}-L\_{\mathrm{foot}}_{x}\right)}_{\mathrm{CF}}$$

where, R_foot is the right foot, L_foot is the left foot, x is the x-value or x-axis coordinate value, PF is the Previous Frame and CP is Current Frame.

Trunk_sway is a measure of how far the subject is bent side to side from the trunk and it is calculated by taking the changes of torso position with respect to the hip position. The amount of bend or the Trunk_sway value is simply an average of the difference of torso and hip position between frames. This variation can be calculated by taking the x-axis values, if the direction of the movement is on the z-axis as shown in the following Equation (5) and using z-axis values instead of x-values if the direction of movement is on the x-axis.

(5) $$\;\mathrm{Trunk}\_\mathrm{sway}\;=\;\frac{{\left({\mathrm{Torso}}_{x}-\left(\frac{L\_{\mathrm{hip}}_{x}+\;R\_{\mathrm{hip}}_{x}}{2}\right)\right)}_{\mathrm{PF}}+{\left({\mathrm{Torso}}_{x}-\left(\frac{L\_{\mathrm{hip}}_{x}+\;R\_{\mathrm{hip}}_{x}}{2}\right)\right)}_{\mathrm{CF}}}{2}$$

where, L_hip is the left hip position and R_hip is the right hip position.

The last parameter of fall risk factors, the Spread_arm is a measure of how much the two arms are spread. This parameter is computed by taking the difference of torso position and the two (left and right) arms. Similar like Trunk_sway, this value is also calculated from the x-axis if the direction of the movement is on the z-axis as shown in Equation (6) and using z-axis values instead of x-values if the direction is on the x-axis. The average of the distance of the two arms to the torso are compared to a threshold between frames to identify any action where the subject is spreading the arms to balance the body or trying to hold something to control the body.

(6) $$\begin{array}{ll} \mathrm{Spread}\_\mathrm{arm}\;\;=\; & {\left(\frac{\left({\mathrm{Torso}}_{x}-R\_{\mathrm{arm}}_{x}\right)+\left({\mathrm{Torso}}_{x}-L\_{\mathrm{arm}}_{x}\right)}{2}\right)}_{\mathrm{CP}}\\ & -{\left(\frac{\left({\mathrm{Torso}}_{x}-R\_{\mathrm{arm}}_{x}\right)+\left({\mathrm{Torso}}_{x}-L\_{\mathrm{arm}}_{x}\right)}{2}\right)}_{\mathrm{PF}} \end{array}$$

where, R_arm is right arm and L_arm is the left arm.

### 3.3. Proposed Fall Detection Algorithm

**Figure 3:**
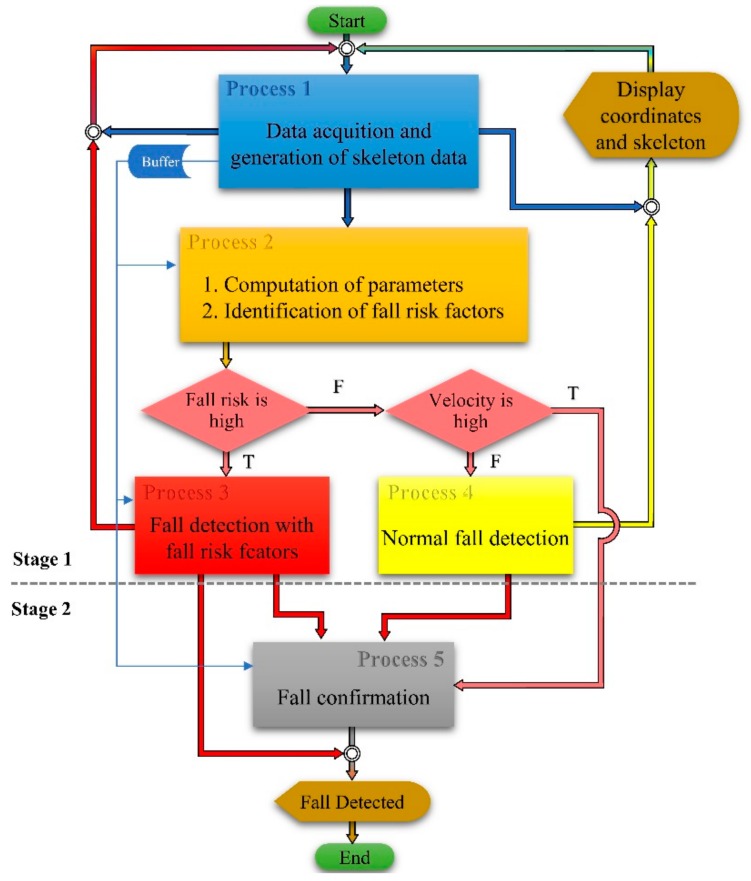
Proposed fall detection algorithm.

The proposed fall detection algorithm primarily consists of two stages for fall detection. The first stage will identify any potential fall event and the second stage will confirm or verify the fall event. These two stages of fall detection are designed into five processes in the proposed fall detection algorithm. The first process checks if a person is detected in the scene from the skeleton data generated by the Kinect sensor. The second process computes the initial height, velocity and fall risk factors. This process can generate a potential fall alert if the initial velocity is high and then pass to Process 5, for fall confirmation. This is by assuming that it is not common to observe a higher velocity from any daily activity for elderly people than for a fall event. Whereas, Process 3, which starts if the fall risk is high, will detect such an alert (potential fall) using another new velocity and height. On the other hand, Process 4, which starts if initial velocity is low, will use height, activity detection and acceleration to identify a potential fall event. These four processes belong to Stage 1 of fall detection and the 5th process will do the fall confirmation or verification of Stage 2, as shown in Figure 3. 

The proposed fall detection algorithm starts from Process 1, which acquires skeleton data from the sensor. The skeleton data are then fed to Process 2, for the computation of the required fall detection parameters; the data is also stored in a buffer, which is available to all other processes. The computed, fall risk factor and velocity from this process, are used to decide the next processes to be executed. If the fall risk factor is flagged as high, Process 3 will be executed to detect the fall with risk factors. In case, if the fall risk factors are normal or low, then the computed velocity from Process 2 will be used to either start Process 4 for normal fall detection or start Process 5, for immediate fall confirmation.

Process 5 is dedicated for fall confirmation if the velocity from Process 2 or Process 3 is flagged as high and no activity or high acceleration is flagged from Process 4. It is also to be noted that the proposed algorithm in Figure 2 immediately executes the fall confirmation process (Process 5) after it notices an abnormal velocity change. But an abnormal increase in velocity can be caused from many activities where the pattern and the changes in direction of height are different, such as an increase in walking speed (increased step frequency or step length), sitting on a chair or floor, lying down on the floor or bed, and running. For the example of walking and running, the height changes are supposed to show a small fluctuation (up and down) on the x-axis and for other activities mentioned above the direction will be straight to the floor or most probably diagonally down to the y-axis of the image.

## 4. Experimental Results and Discussion

**Figure 4:**
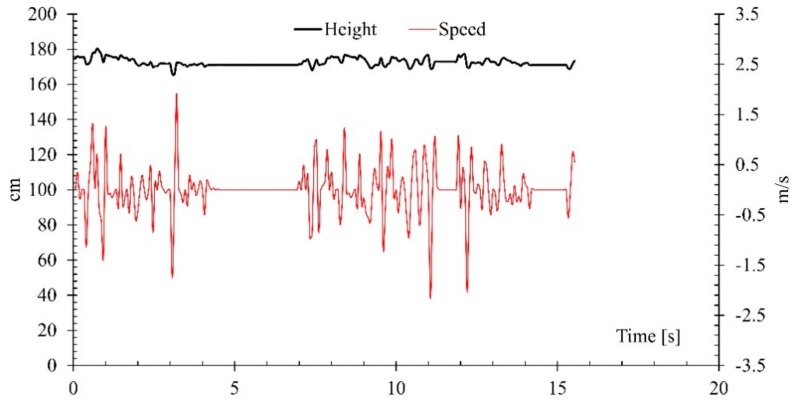
Changes in height with instant walking speed.

The experimental activities conducted showed promising results in adapting the algorithm for the different fall risk levels or simply different simulated walking styles (normal, elderly, and elderly with weak gait). The results include simulations of common daily life activities such as walking in different directions, lying on the floor, picking objects from the floor, sitting on the floor or chair and fall events by two healthy adults in the lab. It is also to be noted that it is not feasible to perform actual measurements of key joints in the home environment [37]; however, the results showed good performance because the proposed algorithm does not require very accurate changes. Rather, it just needs to know rapid changes or the drop of a key joint to floor level. The following Figure 4 shows the changes in height pattern of the distance of the head from the floor for different simulated walking styles including slow, normal and simulated walking with a physically weaker body. 

**Figure 5:**
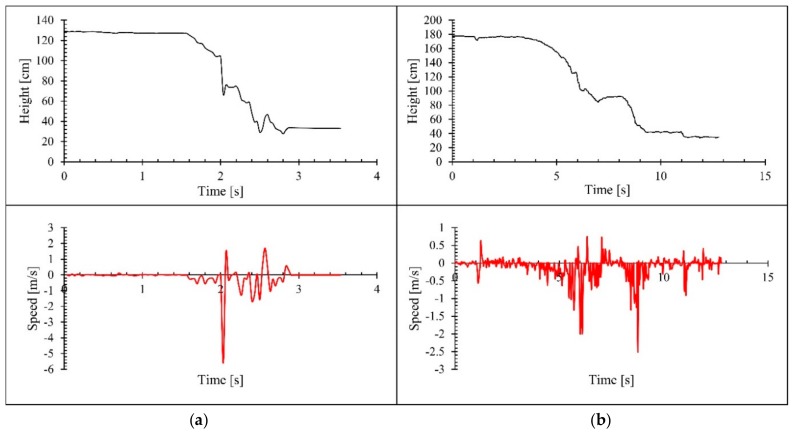
Changes in height and instant speed observed for some fall event. (**a**) Fall while sitting chair; (**b**) fall while trying to sit on floor.

Figure 5 shows the changes in height pattern and instant speed observed for fall events. Part (a) of Figure 5 illustrates a fall event while sitting on a chair and part (b) shows an unintentional fall event while trying to sit on the floor from standing position. The observed instantaneous speed for the changes in the height pattern is evidence for the accuracy of the methods employed to generate the data and applicability of the sensor. 

The proposed fall detection algorithm was also benchmarked on the motion data obtained from the URFD dataset [38]. The dataset contains 70 sequences (30 falls and 40 activities of daily life) in front view. The results of the proposed algorithm on this dataset were compared with results of the study representing the dataset. The experimental results of the related work representing the URFD dataset which used only SVM on the depth data and the results of the proposed algorithm on the URFD data, is compared in this section. The proposed algorithm was run on the acceleration data available with the URFD dataset; the generated results are shown in the following Table 1. The proposed algorithm successfully detected 29 out of the 30 fall events and it failed to classify one sample of fall event. For the other activities of daily life included in the dataset, the proposed algorithm identified 33 activities and failed to classify 7 activities.

The results of the proposed algorithm on the dataset showed relatively lower performance than the results of the related work representing the dataset. The performance of the proposed algorithm on the URFD dataset was also lower than its performance on the simulated activities performed for the evaluations conducted in our lab. The reason is simple, the proposed algorithm was not directly applied on this dataset how it would be in real time simulation, rather it was run on the available acceleration data. The dataset contains depth images or color images and therefore it is very difficult to apply an algorithm which was developed to run in real time. Extraction of information from the already extracted depth image is difficult and will be degraded, then extracting the same information in real time. The depth information also requires 3-dimensional data (3 axis values), which is not possible to extract from an image. Most importantly the data contains acceleration information only and therefore the height information required for the proposed algorithm was computed by extracting the initial height from the depth image and then subtracting the variation in acceleration data for the remaining height change patterns for any activity. Thus, it is very likely to show a varying performance especially when an algorithm which is designed for real time is applied to the extracted information from any such dataset. It is important to note that even with these obstacles the proposed algorithm showed a comparatively close performance to that of the related work representing this dataset. The following Table 2 illustrates the common performance measures of the proposed algorithm on the URFD dataset, with the results of the study [39] representing this dataset.

The proposed algorithm showed relatively lower performance when compared with its performance on our simulated activities; however, it was closely equal to the results of the related work presenting this dataset. The results of the proposed algorithm on this dataset showed higher specificity than the related work, and showed little lower results for the other performance measures. The proposed algorithm failed to identify one real fall event and misclassified seven other activities of daily life. Even though the proposed algorithm was designed to be very sensitive in detecting all likely fall events, the results on this dataset lacked. This is mainly due to the nature of the available data. The available data were very smooth, even for the fall events, but the simulated activities for the evaluation of the proposed algorithm showed higher variations. 

**Figure 6:**
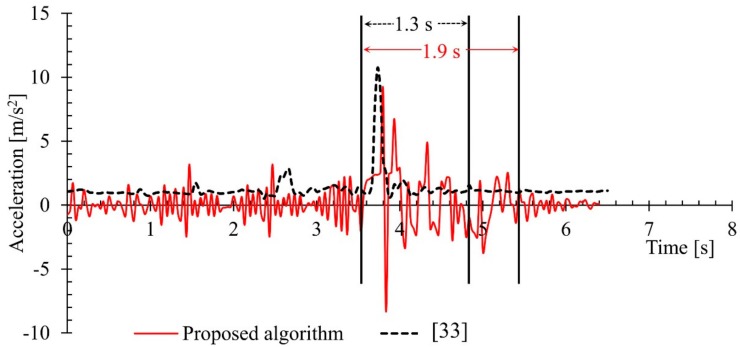
Changes in acceleration for a fall event from the dataset and a similar event from the own simulated activities.

**Figure 7:**
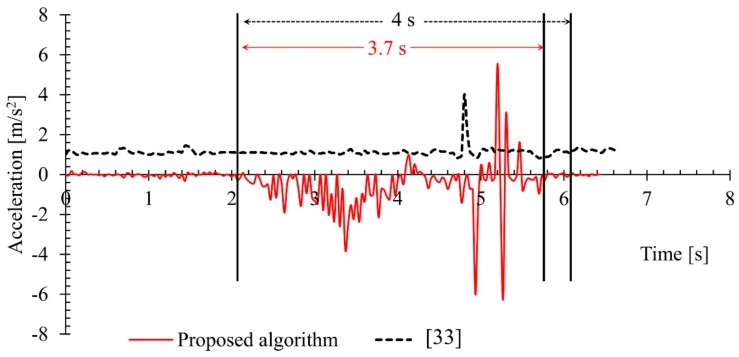
Changes in acceleration for a lying on floor from the dataset and a similar event from the own simulated activities.

The following Figure 6 shows the changes in acceleration of a fall event from the URFD dataset together with a similar fall event from the simulated activities (own dataset) performed in the lab for the evaluation of the proposed algorithm. Figure 7 shows the same changes of acceleration for two subjects lying on floor (one lying on floor from the URFD dataset and the other lying on floor from the own simulated activities) from standing posture. The lines in the red color are representing the changes observed from our own dataset and the dotted lines in the black color are representing the changes observed in the URFD dataset. The data for the two figures are trimmed-off from the beginning to start the two events together.

The fall event shown in Figure 6 is representing the changes in acceleration for a fall event. Similarly, the changes in acceleration for the lying on floor is used to compare with another lying on floor from the dataset, as shown in Figure 7. The fall event from the own simulation took 1.9 s while the fall event taken from the dataset took 1.3 s to completely rest on floor. Lying on floor for the own simulation in the lab took 3.7 s and the same activity taken from the URFD dataset took 4 s to completely rest on floor, as illustrated in Figure 7.

**Figure 8:**
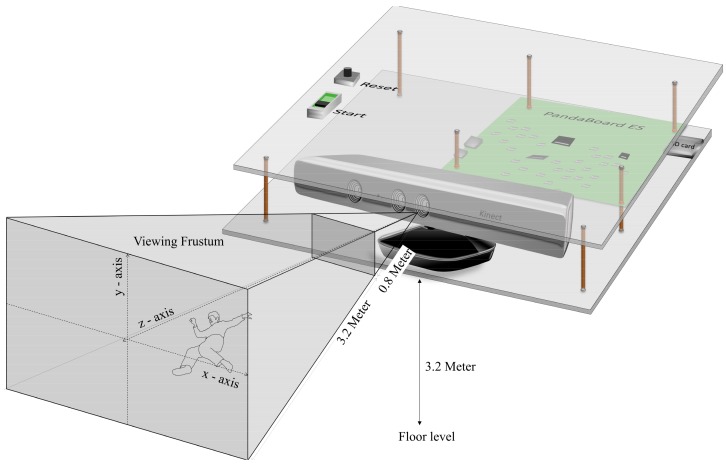
Prototype of the developed system.

It is very clear from the results in Figure 6 and Figure 7, that the data available in the URFD dataset is very smooth, even for the fall event it showed a rapid change only once for about 0.2 s. This would be very challenging for any fall detection algorithm to make a decision from changes in such a small period of time. Thus, it is very likely that the event with such small variations or with less clues will be missed by any algorithm. As a result, the proposed algorithm failed to identify one fall event from this dataset, but it successfully classified all the fall events simulated in the lab (own dataset), as the proposed algorithm was designed to be sensitive. The higher variation observed in own simulation is also evidence that the proposed algorithm can tolerate errors from the loss of information from few frames. This is because the available data from the dataset is very smooth and the proposed algorithm was mainly tested and derived from highly fluctuating data. A prototype of how the developed system looks like and how it should be setup is shown in Figure 8.

## 5. Conclusions

This paper proposed a fall detection algorithm which is adaptable to the user based on the physical strength or the fall risk level at any given time. Fall risk level is a measure of chance of unintentional falls or prediction of falls. The proposed algorithm for fall detection checks the fall risk level at present time before proceeding with fall detection. By this way, the algorithm can adapt the fall detection process to accurately detect fall events from different groups of the population. In order to adapt the algorithm for people with difference fall risk levels, the determinants of normal gait were also studied. The deviation in normal gait (regular walking capability without any aid) were used to predict (fall risk level) falls during fall detection, which accounts for the major contributions of the proposed algorithm. The results showed that the proposed algorithm was able to accurately identify the different fall risk levels from the proposed parameters and thus adapt the fall detection process accordingly. The benchmarking with an available dataset also proved that the proposed algorithm is reliable enough for the application. However, the proposed algorithm was tested on simulated activities in the lab by healthy volunteers which may be very different from real life activities of the elderly. Therefore, future work is still required to test the algorithm on real life activities of elderly and to improve the detection ratio.

## Figures and Tables

**Table 1:** Results of the proposed algorithm on the URFD dataset.

Action/Events	Total	Detected	Missed
Fall events	30	29	1
Other activities	40	33	7

**Table 2:** The results of the proposed algorithm on URFD dataset and the results of the related work representing this dataset.

Performance Measures	[39]	Proposed
Accuracy	90%	88.57%
Sensitivity	100%	96.67%
Specificity	80%	82.5%
Precision	83.3%	80.56%
